# Predictors and Consequences of Global DNA Methylation in Cord Blood and at Three Years

**DOI:** 10.1371/journal.pone.0072824

**Published:** 2013-09-04

**Authors:** Julie B. Herbstman, Shuang Wang, Frederica P. Perera, Sally A. Lederman, Julia Vishnevetsky, Andrew G. Rundle, Lori A. Hoepner, Lirong Qu, Deliang Tang

**Affiliations:** 1 Department of Environmental Health Sciences, Columbia University Mailman School of Public Health, New York, New York, United States of America; 2 Department of Biostatistics, Columbia University Mailman School of Public Health, New York, New York, United States of America; 3 Institute of Human Nutrition, Columbia University Medical Center, New York, New York, United States of America; 4 Department of Epidemiology, Columbia University Mailman School of Public Health, New York, New York, United States of America; University of Bonn, Institut of Experimental Hematology and Transfusion Medicine, Germany

## Abstract

DNA methylation changes have been implicated in many common chronic diseases leading to the hypothesis that environmental and age-related DNA methylation changes within individuals are involved in disease etiology. Few studies have examined DNA methylation changes within an individual over time and all of these studies have been conducted in adults. Here, we aim to characterize how global DNA methylation changes from birth to age three within a longitudinal birth cohort study and to determine whether there are consistent predictors of DNA methylation levels measured three years apart. We measured global DNA methylation in the same children at birth (cord blood) and again at three years of age among 165 children, using an immunoassay. We found that on average, DNA methylation was significantly higher in blood at age 3-years than in cord blood (p<0.01). However, for any individual child, the difference was less than would be expected by chance. We found that pre-pregnancy BMI was negatively predictive of both cord and three-year DNA methylation, even after statistical adjustment to account for the correlation between cord blood and three-year DNA methylation. The biologic implications of small changes in global DNA methylation are unknown. However, the observation that global DNA methylation levels persist within an individual from birth to age three supports the belief that factors that influence global DNA methylation, including pre-pregnancy BMI, may confer long-term effects.

## Introduction

DNA methylation is an epigenetic process by which non-sequence-based regulatory information can be mitotically transferred from mother to daughter cell. Involving the addition of a methyl group at the 5-position of a cytosine-guanine (CpG) dinucleotide, DNA methylation inhibits gene expression by interfering with transcription binding proteins and is associated with chromatin remodeling [1]. CpG sites are not evenly represented throughout the genome. Over ninety percent of methylated CpG sites lie within transposable, repetitive elements, resulting in their silenced expression [2], [3]. Other clusters occur in gene promoters.

DNA methylation patterns are reset during early embryogenesis, allowing for tissue differentiation. Thereafter, DNA methylation is thought to be conserved, albeit labile, in somatic cells, allowing the organism to adjust to changing developmental and environmental conditions [1], [4], [5]. DNA methylation changes have been implicated in many common chronic diseases leading to the hypothesis that environmental and age-related DNA methylation changes within individuals are involved in disease etiology [6]. Therefore, understanding how DNA methylation changes within an individual over time may provide important links for etiological research.

Relatively few studies have examined DNA methylation changes within an individual over time and all of these studies have been conducted in adults [3], [7], [8]. Given the theory that many adult chronic diseases have developmental origins [9], [10] and that DNA methylation may be most vulnerable to insult when it is undergoing developmental reprogramming, it has been suggested that epigenetic mechanisms might be involved in the developmental origins of health and disease [6]. Therefore, understanding how DNA methylation changes early in life could provide critical information linking prenatal exposure and subsequent disease or dysfunction. Understanding factors that predict as well as those that result from DNA methylation patterns early in life can provide clues about disease etiology. For example, previous work by Michels et al. indicates that maternal pre-pregnancy body mass index (BMI) is associated with global DNA methylation measured at birth, which is, in turn, associated with birth weight [11].

In this report, we aim to characterize how global DNA methylation changes from birth to age three within our longitudinal birth cohort study and to determine whether there are consistent predictors of DNA methylation levels measured three years apart. Additionally, we sought to confirm the findings concerning DNA methylation, pre-pregnancy BMI and birth weight reported previously by Michels et al. Further, given the theory that DNA methylation levels may persist throughout childhood, we sought to determine whether maternal pre-pregnancy BMI is also associated with global DNA methylation measured in the offspring at three years of age.

## Methods

### Study Population

The Northern Manhattan Mothers and Newborns Study of the Columbia Center for Children’s Environmental Health (CCCEH) is a longitudinal cohort study of African American and Dominican women who were recruited in the prenatal clinics of New York-Presbyterian Medical Center, Harlem Hospital or their satellite clinics, as previously described [12]. Eligible women were those who did not smoke or use illicit drugs, aged 18–35 at delivery, registered in the prenatal clinics by the 20^th^ week of pregnancy, free of reported diabetes, hypertension and HIV, and had resided in the Washington Heights, Central Harlem or the South Bronx areas of New York City for at least one year. Eligible women who gave informed consent were considered ‘initially enrolled’. Women who completed the prenatal questionnaire and provided maternal and/or umbilical cord blood were considered ‘fully enrolled’ [13]. For this study, we investigated n = 279 children with available data on umbilical cord blood DNA that had been isolated from total white blood cells (WBC). Among these children, 165 also had DNA from blood collected at three years of age. Participants with cord blood DNA methylation measurements were not significantly different from the underlying cohort with respect to demographic characteristics; however, among those with cord blood DNA methylation measurements, children with three year measurements had mothers with greater average weight gain during pregnancy (36.5 lbs vs. 30.9 lbs) **(Table S1 in File S1)**. All study participants provided their written informed consent. In addition, the women enrolled in this study provided consent on behalf of their children, who at age 3 were too young to provide assent. This protocol and related informed consent procedure is approved by the Columbia University Medical Center Institutional Review Board.

### DNA methylation

DNA isolated from umbilical cord blood leukocytes (WBC) was analyzed for global DNA methylation using the Methylamp^TM^ Global DNA Methylation Quantification Kit (Epigentek Group Inc, NY). This method quantifies the methylated fraction of DNA using an ELISA-like reaction. The proportion of methylated DNA in the full sample is determined by plotting the intensity of the optical density generated from the reaction in comparison to the linear range of a standard curve produced using a methylated DNA control. Using this assay, DNA methylation is represented as the ratio of methylated DNA per 100 nanograms (ng) of total DNA. In humans, with the exception of stem cells, which make up a very small proportion of cord blood, DNA methylation occurs exclusively at cytosine-guanine dinucleotides [14], [15]. As CpG dinucleotides are underrepresented in the genome, the proportion of total DNA that would be expected to be methylated is small. Reference values provided by the manufacturer range from 0.8–3%. Samples were run in duplicate, and cord and three year samples from the same children were run on the same reaction plate to minimize the plate-to-plate variation. The average of the duplicate measures was used in analyses. In this sample, the coefficient of variation (calculated using the method described in [16]) between replicates on the same plate was 16%.

### Birth outcomes and covariates

Research workers abstracted information about birth outcomes including gestational age at delivery, birth weight, birth length, and head circumference from maternal and infant medical records after delivery. We also collected additional information about important covariates including infant sex, maternal height, pre-pregnancy weight, total gestational weight gain (weight closest to delivery minus pre-pregnancy weight), and complications of pregnancy and delivery. Standardized pre-pregnancy body mass index (BMI) categories were grouped according to the recommendations of the CDC (2011) as underweight, normal weight, overweight and obese [17], using maternal height (as measured by research workers) and pre-pregnancy weight (self-reported). Bilingual (Spanish and English) research workers administered a 45-minute questionnaire during the last trimester of pregnancy. This questionnaire included demographic information, history of active and passive smoking, and socioeconomic information related to income and education. A follow-up questionnaire at 6 months, 1, 2, and 3 years provided additional information about breast feeding duration and postnatal environmental tobacco smoke exposure (ETS). Prenatal exposure to environmental polycyclic aromatic hydrocarbons (PAH) was assessed using a backpack monitor for 48 hours during the second trimester of pregnancy, as previously described in detail [12], [13]. We also used the woman’s questionnaire-based report of smoking in the home at three years to account for postnatal exposure to passive smoke.

### Statistical analyses

Descriptive statistics were calculated for DNA methylation in cord and three-year blood samples. Cord and three-year DNA methylation levels were initially compared statistically using Pearson’s and Spearman correlation coefficients. To test the hypothesis that the difference between DNA methylation levels in cord and three-year samples from the same individuals was significantly smaller than the difference between DNA methylation levels in cord and three-year samples from different individuals, we conducted permutation analyses. More specifically, with 165 children that have both cord and three-year DNA methylation samples, we held the cord DNA methylation data intact and shuffled the three-year DNA methylation to generate cord and three-year pairing from different individuals, which we call random pairs. We repeated the permutation 10,000 times, which generated a distribution of the average difference in DNA methylation between 165 randomly paired cord and three-year samples from different children. We then compared the average difference in DNA methylation between the 165 observed cord and three-year pairs from the same individuals to the 10,000 average differences in DNA methylation between the 165 randomly paired cord and three-year samples from different individuals.

Univariate associations between demographic, socioeconomic, and other potential covariates and cord and three-year DNA methylation, respectively, were explored using Student’s t-test and simple linear regression, as appropriate. In linear regressions, DNA methylation measures were natural log-transformed to meet normality assumptions. Change in DNA methylation was calculated as the absolute difference in DNA methylation from birth to three years. To further determine which factors predicted cord and three-year DNA methylation or the change in DNA methylation over time, respectively, we used multiple linear regression models where natural log-transformed DNA methylation (either cord or three-year or the change in DNA methylation) was the dependent variable. In the model where three-year natural log-transformed DNA methylation was the dependent variable, we constructed a separate model that included cord natural log-transformed DNA methylation as a covariate to determine the independent influence of other covariates on DNA methylation measured at three years. We used principal components analysis to deconstruct the variation associated with plate-to-plate variation associated with technical variability [18], [19]. We then used the single factor variable that accounted for 98% of the variation across plates as a covariate in all analyses other than the cord-three year DNA methylation comparisons which were matched on plate by design.

Finally, we evaluated the relationships between cord blood DNA methylation and birth outcomes, including gestational age at delivery, birth weight, length, head circumference and ponderal index (*birth weight in g/(length in cm)^3^ x 100*), a measure of body proportions at birth. Global DNA methylation measured in cord blood, an independent variable, was log transformed to stabilize the variance. Covariates were included in the birth outcome models if they were associated with the outcome in bivariate analysis with p<0.10 and changed the beta coefficient representing the relationship between cord blood DNA methylation and each birth outcome more than 5%.

## Results

**Figure 1:**
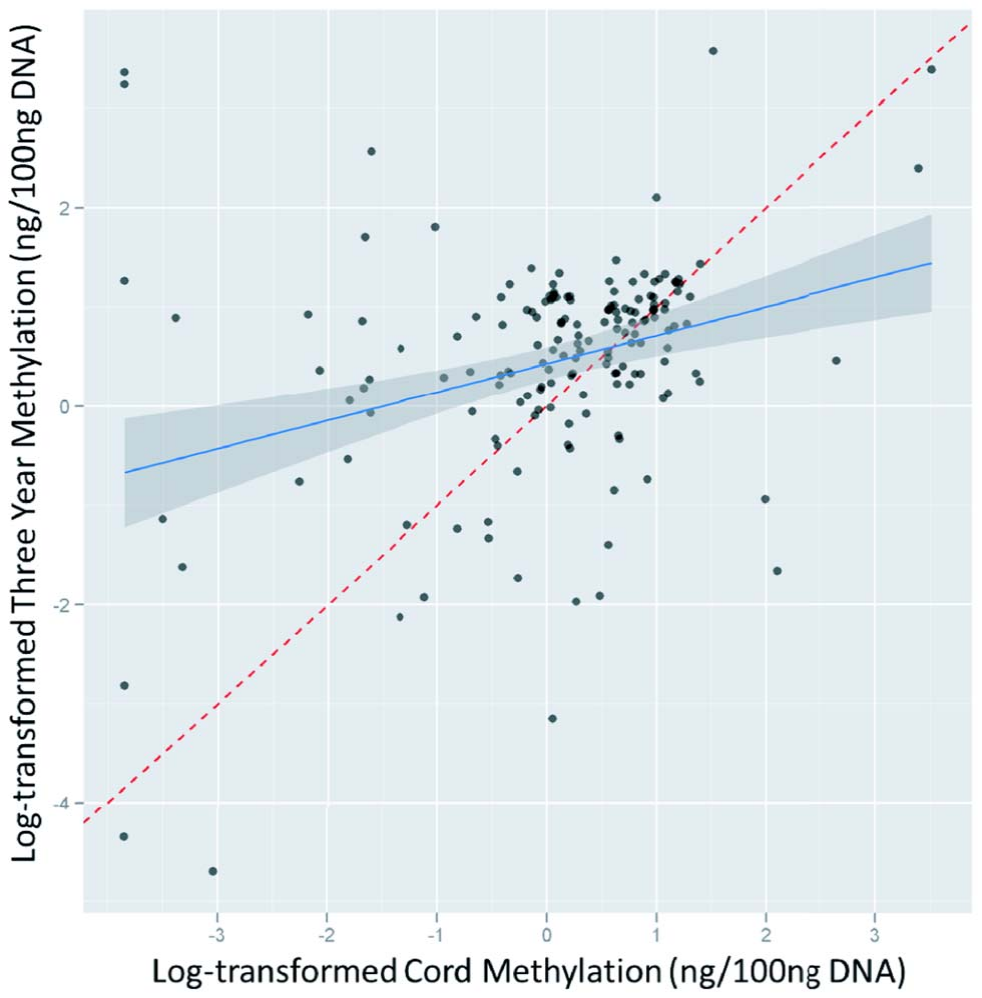
Association between cord and three-year DNA methylation.

The mean of cord blood DNA methylation is 1.77 ng/100 ng total DNA (1.84 ng/100 ng total DNA in the subset of participants for whom we also have DNA at three years, “subset”), **Table 1**. Compared to cord blood, average peripheral blood DNA methylation is significantly higher in the same children at age 3 (mean at 3 years: 2.72 ng/100 ng total DNA, paired t-test: p<0.01). Cord and three-year DNA methylation are moderately but significantly correlated (Pearson’s R = 0.41 (p<0.001), Spearman R = 0.30 (p<0.001)), **Figure 1**. Cord DNA methylation is a significant predictor of three-year DNA methylation (β = 0.27, p<0.001 per increase in log-transformed cord DNA methylation).

**Table 1:** Summary Statistics.

	Cord DNA Methylation(ng/100ng total DNA)		Cord DNA Methylation (subset^a^)(ng/100ng total DNA)		DNA Methylation at 3 yrs(ng/100ng total DNA)	
N	279		165		165	
Mean (SD)	1.77	(2.37)	1.84	(2.93)	2.72	(4.58)
Geometric mean	1.13	(3.14)	1.13	(3.60)	1.55	(3.19)
1^st^ quartile	0.84		0.72		1.14	
Median	1.34		1.27		1.94	
3^rd^ quartile	2.18		2.23		2.87	
Spearman R (p-value)					0.30	(<0.01)
Pearson’s R (p-value)					0.41	(<0.01)

Summary Statistics, where subset refers to those with both cord and three year DNA methylation measures available.

a The ‘subset’ includes children who have contributed both cord and three year DNA samples.

**Figure 2:**
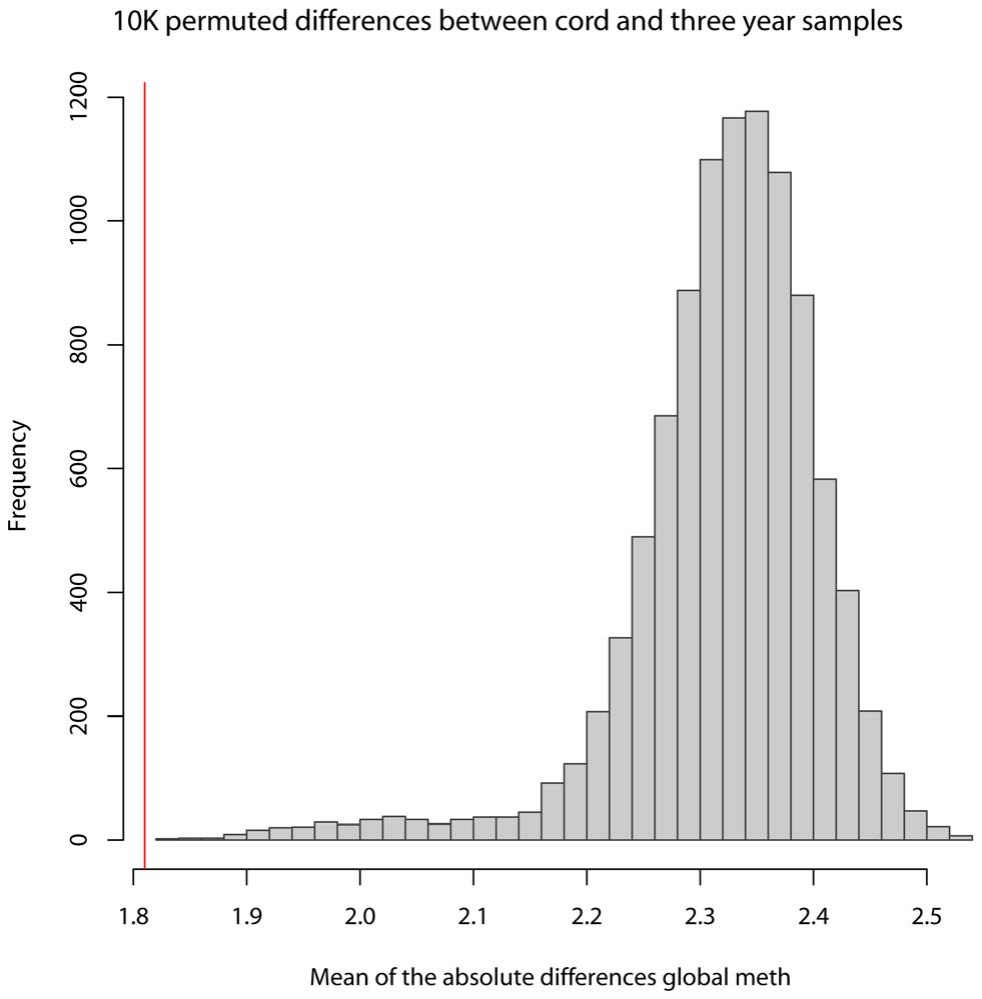
Permutation analysis. Gray histogram depicts the distribution of absolute mean differences in DNA methylation from the 10,000 random pairings of cord and three-year samples. The solid red bar indicates the actual mean absolute difference in DNA methylation from birth to age three in the same individual.

The average change in DNA methylation measured in cord blood and again in blood at age three in the same individuals was 0.88 ng/100ng total DNA (standard deviation  = 4.32). The range was −12.62 to 31.11, such that two-thirds of individuals had global DNA methylation that increased from cord to three years and one-third of individuals had DNA methylation that decreased during this same time period. However, the majority of individuals (62%) had DNA methylation that changed very little (within 1 ng/100ng total DNA) from birth to age 3 while 12% had a negative change greater than 1 ng/100 ng total DNA and 27% had a positive change greater than 1 ng/100 ng total DNA). In the permutation analyses to assess whether the difference between DNA methylation levels in cord and three-year samples from the same individuals were significantly smaller than the difference between DNA methylation levels in cord and three-year samples from different individuals, we compared the 10,000 permuted mean difference from random pairs and one mean difference from the observed pairs. **Figure 2** shows the distribution of absolute mean differences in DNA methylation from the 10,000 random pairings of cord and three-year samples. The solid red bar indicates the actual mean absolute difference in DNA methylation from birth to age three from the same individual. The averaged absolute mean difference between random cord and three-year pairs over 10,000 permutations was 2.33 ng/100 ng total DNA, which was significantly greater than the observed absolute mean difference (1.81 ng/100ng total DNA, (p<0.01)).

The factors that predict cord and three year DNA methylation are shown in **Tables 2** **and** **3**. In univariate analyses after accounting for inter-plate variation, only pre-pregnancy weight and pre-pregnancy BMI predicted lower global DNA methylation in cord blood. Pre-pregnancy BMI was inversely associated with DNA methylation (cord p = 0.03, subset p<0.01, and three-year p = <0.01). When examining BMI group, women who were obese prior to pregnancy had the lowest DNA methylation (compared to women of normal weight: full p = 0.15, subset p = 0.04, and three-year p<0.01).

**Table 2:** Univariate a Predictors of Cord and Three Year DNA Methylation (categorical).

	Cord DNA Methylation (ng/100ng total DNA)				Cord and Three Year DNA Methylation (ng/100ng total DNA)					
							Cord (subset) b		Three Year	
	N	%	Mean	SD	N	%	Mean	SD	Mean	SD
**Maternal Education**										
<HS	100	36.0	1.03	1.12	55	33.3	0.90	1.17	1.48	1.16
HS	96	34.5	1.17	1.12	67	40.6	1.18	1.15	1.87	1.15
>HS	82	29.5	1.20	1.13	43	26.1	1.00	1.20	1.22	1.19
**Marital Status**										
Married/Cohab.	206	75.2	1.04	1.15	119	73.0	0.94	1.19	1.56	1.19
Unmarried	68	24.8	1.15	1.08	44	27.0	1.05	1.11	1.52	1.11
**Material Hardship**										
No	159	57.0	1.07	1.09	93	56.4	0.97	1.13	1.60	1.12
Yes	120	43.0	1.21	1.11	72	43.6	1.13	1.15	1.48	1.14
**Public Assistance**										
No	23	8.3	1.14	1.26	10	6.1	1.60	1.45	1.58	1.43
Yes	255	91.7	1.12	1.07	155	93.9	1.00	1.10	1.54	1.09
**Ethnicity**										
Dominican	193	69.2	1.16	1.08	111	67.3	1.06	1.12	1.68	1.11
African American	86	30.8	1.06	1.13	54	32.7	0.98	1.17	1.30	1.17
**BMI**										
Underweight	14	5.0	1.19	1.35	9	5.5	1.05	1.47	1.49	1.43
Normal weight	125	44.8	1.28	1.10	74	44.8	1.31	1.14	2.01	1.13
Overweight	62	22.2	0.97	1.15	40	24.2	0.84	1.20	1.85	1.18
Obese	78	28.0	1.02	1.13	42	25.5	0.82	1.20	0.83	1.18
**Nulliparous**										
No	137	49.5	1.21	1.10	79	48.17	1.16	1.14	1.68	1.14
Yes	140	50.5	1.04	1.10	85	51.83	0.91	1.14	1.42	1.13
**Former smoker**										
No	212	81.9	1.12	1.08	126	82.9	1.07	1.11	1.78	1.11
Yes	47	18.1	1.18	1.18	26	17.1	0.85	1.26	1.00	1.25
**ETS**										
No	181	66.1	1.11	1.09	104	64.2	0.99	1.12	1.63	1.12
Yes	93	33.9	1.15	1.12	58	35.8	1.12	1.17	1.46	1.16
**Sex**										
Male	130	46.6	1.14	1.10	78	47.3	0.96	1.14	1.50	1.14
Female	149	53.4	1.12	1.10	87	52.7	1.10	1.13	1.59	1.13

Univariate^ a^ Categorical Predictors of Cord and Three Year DNA Methylation, geometric mean and SD.

a Analyses corrected for inter-plate variation.

b The ‘subset’ includes children who have contributed both cord and three year DNA samples.

Abbreviations: Yr (year); HS (High School); BMI (Body Mass Index); ETS (Environmental Tobacco Smoke).

**Table 3:** Univariate a Predictors of Cord and Three Year DNA Methylation (continuous).

		Cord DNA Methylation (ng/100ng total DNA)				Cord DNA Methylation (subset)^b^ (ng/100ng total DNA)			Three Yr DNA Methylation (ng/100ng total DNA)	
	N	Statistical estimate		p- value	N	Statistical estimate		p-value	Statistical estimate	p-value
Maternal Ht	277	**beta**	−0.003	0.889	165	**beta**	0.020	0.487	−0.035	0.194
		**rho**	−0.018	0.770		**rho**	0.045	0.570	−0.066	0.400
Pre-preg. Wt.	270	**beta**	−0.004	0.018	159	**beta**	−0.005	0.055	−0.009	0.000
		**rho**	−0.125	0.040		**rho**	−0.199	0.012	−0.315	0.000
Pre-preg. BMI	269	**beta**	−0.023	0.029	159	**beta**	−0.033	0.027	−0.049	0.000
		**rho**	−0.124	0.043		**rho**	−0.234	0.003	−0.293	0.000
Weight gain	252	**beta**	0.003	0.433	152	**beta**	0.007	0.265	0.000	0.986
		**rho**	0.050	0.434		**rho**	0.037	0.651	−0.086	0.293
Maternal age	279	**beta**	−0.005	0.743	165	**beta**	−0.023	0.229	−0.038	0.040
		**rho**	−0.040	0.502		**rho**	−0.184	0.018	−0.176	0.023
Total PAH	268	**beta**	0.076	0.361	159	**beta**	0.080	0.524	−0.067	0.576
		**rho**	−0.03	0.66		**rho**	0.076	0.344	−0.095	0.235

Univariate^ a^ Continuous Predictors of Log-transformed Cord and Three Year DNA Methylation, Beta and Correlation Coefficients.

a Regression analyses corrected for inter-plate variation.

b The ‘subset’ includes children who have contributed both cord and three year DNA samples.

Abbreviations: Yr (year); Ht (height); Wt (weight); BMI (Body Mass Index); PAH (Polycyclic Aromatic Hydrocarbons).

At three years, we found that having a smoker in the home at three years was (non-significantly) associated with lower three year DNA methylation (p = 0.08) and having breast fed more than 3****months was (non-significantly) associated with higher three year DNA methylation (p = 0.07) compared with not having breast fed at all. Previously, we reported an association between prenatal PAH and lower cord blood DNA methylation [20]. Among those for whom we measured three year DNA methylation, high PAH (above the population median) was not associated with lower DNA methylation at three years.

In multiple linear regression models (**Table 4**), pre-pregnancy BMI was significantly predictive of both cord and three year DNA methylation (full p = 0.01, subset p = 0.02, three-year p<0.01). Because we observed a significant positive association between cord and three year DNA methylation (**Table 1**, **Figures 1** and **2**), we explored whether cord blood DNA methylation was an independent predictor of three year DNA methylation or whether there is evidence indicating that it is on the causal pathway between pre-pregnancy BMI and three year DNA methylation. Adjusting for other covariates listed in **Table 4**, the beta coefficient for pre-pregnancy BMI on log-transformed DNA methylation at three years was −0.069, with a 95% CI of −0.102, −0.036. After further adjusting for log-transformed cord DNA methylation, the coefficient for pre-pregnancy BMI was virtually unchanged (−0.062, 95% CI: −0.096, −0.029), indicating that cord methylation does not confound the observed association between pre-pregnancy BMI and three year methylation. In **Figure S1A-D in File S1**, we present the partial regression plots (reflecting the regression coefficient from the multiple linear regression models) presenting the association of pre-pregnancy BMI and DNA methylation in cord and three-year DNA. Being a former smoker and concurrent ETS exposure (defined as having a smoker in the home at three years) were also predictors of lower global DNA methylation in the offspring at three years (p = 0.10 and p = 0.08, respectively).

**Table 4:** Multivariate a Predictors of Cord and Three Year DNA Methylation.

	Cord DNA methylation (N = 237) (ng/100ng total DNA)			Cord DNA methylation (subset) (n = 139)^b^ (ng/100ng total DNA)			Three year DNA methylation (n = 139) (ng/100ng total DNA)			Three year DNA methylation, adj for cord methylation (N = 139) (ng/100ng total DNA)		
	Beta	95% CI		Beta	95% CI		Beta	95% CI		Beta	95% CI	
**Maternal Education**												
HS (vs. no HS)	0.103	−0.291,	0.496	0.301	−0.196,	0.798	0.033	−0.412,	0.478	−0.005	−0.448,	0.439
>HS (vs. no HS)	0.090	−0.328,	0.509	0.077	−0.498,	0.652	−0.281	−0.795,	0.233	−0.299	−0.809,	0.211
**Married (vs. unmarried)**	−0.190	−0.604,	0.224	−0.275	−0.836,	0.287	0.135	−0.368,	0.638	0.163	−0.336,	0.663
**Material Hardship**	0.094	−0.234,	0.421	0.091	−0.349,	0.530	−0.191	−0.584,	0.202	−0.199	−0.589,	0.191
**Afr. Am. (vs. Dominican)**	−0.030	−0.395,	0.334	0.052	−0.473,	0.576	−0.052	−0.532,	0.427	−0.073	−0.549,	0.402
**Prenatal ETS**	−0.068	−0.435,	0.298	0.046	−0.458,	0.549	0.247	−0.239,	0.733	0.215	−0.268,	0.698
**Former smoker**	0.010	−0.355,	0.374	−0.304	−0.829,	0.220	−0.470	−0.949,	0.009	−0.437	−0.913,	0.038
**Male (vs. female)**	−0.036	−0.356,	0.284	−0.280	−0.708,	0.148	−0.265	−0.650,	0.120	−0.227	−0.611,	0.158
**Multiparous (vs. nulliparous)**	−0.060	−0.435,	0.314	−0.020	−0.543,	0.503	0.099	−0.365,	0.564	0.107	−0.354,	0.568
**Pre-pregnancy BMI**	−0.035	−0.062,	−0.007	−0.044	−0.081,	−0.008	−0.069	−0.102,	−0.036	−0.062	−0.096,	−0.029
**Weight Gain**	0.000	−0.010,	0.011	0.003	−0.012,	0.018	−0.010	−0.023,	0.004	−0.010	−0.023,	0.004
**Maternal Age**	−0.011	−0.053,	0.031	−0.032	−0.087,	0.024	−0.044	−0.094,	0.006	−0.038	−0.088,	0.013
**Total PAH (log-adj)**	0.085	−0.113,	0.282	0.070	−0.231,	0.370	0.137	−0.133,	0.407	0.124	−0.145,	0.392
**Breast Fed**							0.008	−0.012,	0.027	0.006	−0.014,	0.025
**ETS at 3 yrs**							−0.551	−1.072,	−0.030	−0.470	−0.994,	0.055
**Cord DNA methylation**										0.146	−0.018,	0.309
**R^2^**	0.0979			0.274			0.304			0.322		
**Adjusted R^2^**	0.0363			0.188			0.207			0.221		

Multivariate^a^ Predictors of Cord and Three Year DNA Methylation.

a All multivariate models corrected for inter-plate variation.

b The ‘subset’ includes children who have contributed both cord and three year DNA samples.

Abbreviations: adj (adjusted); HS (high school); Afr. Am. (African-American); ETS (Environmental Tobacco Smoke); BMI (Body Mass Index); PAH (Polycyclic Aromatic Hydrocarbons).

We explored the factors that influence the absolute value of the difference between DNA methylation measured in cord blood and DNA methylation measured in blood collected from the same children at age 3 (**Table S2 in File S1** ). Pre-pregnancy BMI predicted a smaller absolute change in DNA methylation from birth to three years. Breast feeding duration during the first year was associated with a larger absolute change in DNA methylation.

In **Table 5**, we present the univariate and adjusted association between cord DNA methylation and birth outcomes including gestational age, birth weight, birth length, ponderal index, and head circumference. In no case was cord DNA methylation significantly associated with any of these birth outcomes, either before or after adjusting for possible confounders.

**Table 5:** Association with Birth Outcomes a.

		Model 1 b			Model 2 c			Model 3 d		
	N	Beta	95% CI		Beta	95% CI		Beta	95% CI	
**Gestational Age**	252	0.020	−0.114,	0.154	0.025	−0.109,	0.159	0.022	−0.113,	0.156
**Birth Weight**	249	−36.736	−80.868,	7.396	−25.679	−69.404,	18.047	−28.452	−72.064,	15.161
**Birth Length**	236	−0.008	−0.306,	0.290	0.048	−0.250,	0.346	0.049	−0.248,	0.347
**Ponderal Index**	237	−0.213	−0.643,	0.217	−0.283	−0.695,	0.130	−0.274	−0.688,	0.140
**Head Circumference** e	230	−0.154	−0.296,	−0.012	−0.120	−0.256,	0.016	−0.128	−0.263,	0.006

Cord DNA Methylation (ng/100ng total DNA) and Birth Outcomes^ a^.

a All multivariate models corrected for inter-plate variation.

b Model 1 adjusted for gestational age (except in the model where gestational age is the dependent variable) and plate.

c Model 2 adjusted for covariates in Model 1 plus maternal height, pre-pregnancy BMI, maternal age at delivery, ethnicity, sex, and public assistance.

d Model 3 adjusted for covariates in Model 2 plus total Polycyclic Aromatic Hydrocarbons and Environmental Tobacco Smoke.

e Delivery mode added to all models where head circumference is the dependent variable.

## Discussion

The goals of this analysis were three-fold. First, we explored the relationship between global DNA methylation measured in the same children at birth (cord blood) and again at three years of age. We found that on average, DNA methylation was significantly higher in blood at age 3****years than in cord blood. However, for any individual child, the difference was less than would be expected by chance. Second, we explored the factors that predict global DNA methylation at birth and three years. We found that pre-pregnancy BMI was negatively predictive of both cord and three-year DNA methylation, even after statistical adjustment to account for the correlation between cord blood and three-year DNA methylation. Finally, we examined whether global DNA methylation in cord blood is a risk factor for adverse birth outcomes. We did not find any evidence that cord blood DNA methylation was associated (either positively or negatively) with birth outcomes.

Changes in epigenetic marks have been proposed to be a biologic mechanism by which exposures affect health and disease across the life course [6]. Generally, epigenetic marks are thought to be conserved and perpetuated in daughter cells, thereby predisposing cells towards an altered gene expression when the affected genes are developmentally signaled [21]. However, there may be some alterations in epigenetic marks that are more transient. If these changes occur during developmentally important periods, they may trigger a biologic cascade thereby influencing downstream health. Thus far, there has been little documentation characterizing the persistent or transient nature of DNA methylation during early child development.

Because global DNA hypomethylation is a hallmark of many age-related diseases [22]–[24] a number of studies have examined changes in DNA methylation over time in older populations [3], [7], [8]. Bollati et al. reported a gradual decrease in DNA methylation of Alu (but not LINE-1) measured in WBC by pyrosequencing repetitive elements assessed an average of 8 years apart among men between the ages of 55 and 92. These changes over time were noted to be small relative to the inter-individual variability of global DNA methylation [3]. Bjornnson et al. studied individuals aged 69–96 whose global DNA methylation was measured in WBC 11 years apart. They found that average DNA methylation was equally likely to increase or decrease. Among approximately one third of the individuals studied, DNA methylation changed more than 10%. Using a different population of individuals clustered within families whose WBC global DNA methylation was measured twice over an average of 16 years, Bjornnson et al. showed that DNA methylation change over time tended to cluster within families not necessarily living in the same household. These data suggest that global DNA methylation maintenance is a heritable trait [7]. Wu et al. described global DNA methylation in peripheral blood mononuclear cells (a subset of total WBC) measured three ways (LUMA, LINE-1 by pyrosequencing and Sat2 by methylite) in adults at two time points 8.6 years apart. Using LUMA, the results were similar to those of Bjornnson et al.; approximately 30% had DNA methylation changes greater than 10% compared to 60% using Sat2 and 4% using Line-1 [8]. While all of these analytic methods are considered surrogate measures of global DNA methylation, each measures a different underlying biologic construct, which may explain the discrepant findings.

Here, we report changes in global DNA methylation measured at birth and age three. This is an important life stage to examine, as DNA methylation marks may be programmed during development – a fluid process that does not end at birth – and maintained thereafter. Because much of development, including tissue differentiation, is epigenetically driven [21], factors that influence epigenetic marks and potential plasticity during this period of flux may be especially important. The significant relation of maternal pre-pregnancy BMI and maternal age at delivery to DNA methylation at birth and at three years may be an example of this phenomenon. In addition, it is known that the composition of lymphocytes differs between cord and peripheral blood [25] and DNA methylation differs by cell type [8]. On average, the majority of leukocytes at birth are neutrophils (61%), followed by lymphocytes (31%), monocytes (6%), and eosinophils (2%). At age 2–4 years, the lymphocytes dominate (50–59%), followed by neutrophils (33–42%), monocytes (5%), and eosinophils (2%) [26]. A recent report by Reinius et al. indicates that methylation at CpG sites measured on the Illumina Infinium Human Methylation 450K chip tend to differ by cell lineage [27]. However, only a quarter of the CpG sites represented on the Infinium array represent intragenic regions [28]. Because the majority of methylated CpG sites are located in intergenic regions to silence transposable, repetitive elements, it is not clear that cell-specific changes in DNA methylation on CpG sites located in promotors or gene bodies would have a profound impact on a measure of global DNA methylation [2]. It may be reasonable to speculate that intergenic regions would be preferentially silenced irrespective of cell type. However, because DNA methylation in this study was measured in total blood leukocytes (WBC), we cannot rule out the possibility that the compositional change from birth to three years may explain the average increase in DNA methylation we observed. Unfortunately, differential blood counts were not available in this study to further explore this issue. The intra-individual change in DNA methylation between birth and three years that we observed was, overall, fairly small compared to the inter-individual differences. Similar to the report by Wu et al., the higher the DNA methylation level we observed at baseline (i.e., at birth), the smaller the change in DNA methylation measured at three years.

The observed protracted relation of pre-pregnancy BMI to global DNA methylation measured in both cord and three-year blood in the offspring is of particular note. A sub-optimal intrauterine environment resulting from maternal obesity could predispose the newborn infant and child to a variety of adverse health outcomes [29], [30]. Obesity constitutes a chronic state of low-grade inflammation, which is a critical risk factor for insulin resistance and other metabolic disorders [31]. Additionally, maternal obesity is likely to be related, at least in part, to maternal nutritional factors. Both chronic inflammation [32] and maternal nutrition [33] have been associated with changes in epigenetic markers. Currently, it is not known how maternal obesity prior to pregnancy may change DNA methylation in the offspring and it is also not known whether or what type of risk this change confers on the health of the offspring over his/her life course. We observed associations between pre-pregnancy BMI and DNA methylation at three years that occurred independently of the apparent maintenance of DNA methylation.

The World Health Organization has estimated that one third of U.S. women of reproductive age (20 to 44 years) are obese [34] and obesity rates assessed at the start of pregnancy in the Pregnancy Risk Assessment Monitoring System (PRAMS) have increased 70% from 1993 to 2003 [35]. Given the prevalence of pre-pregnancy obesity and its apparent association with epigenetic modification at birth and during childhood, additional research confirming this association and understanding the health effects conferred are warranted.

In contrast to the report by Michels et al. [11], we did not find an association between global DNA methylation measured in cord blood and birth weight (or any other birth outcome). A number of differences in study design may account for the discordant findings – most notably, the method used to assess global DNA methylation. Michels et al. estimated global DNA methylation using LINE-1 by pyrosequencing in contrast to our immunoprecipitation-based assay. As Wu et al. demonstrated [8], not all measures of global DNA methylation estimate the same biologic construct, despite the fact that they purport to estimate global DNA methylation. Differences in the method used to approximate global DNA methylation may alone account for differences in the associations observed. Additionally, Michels et al. characterized birth weight using standardized categories for low (<2500g) and high (>4000g) birth weight. In this sample, we do not have enough children falling in these categories to analyze our data in a similar fashion. Birth weight, itself, is not a mechanism but rather a biomarker representing, among other things, the degree of restriction of the intrauterine environment [36]. If DNA methylation and birth weight are related, it may only be evident when comparing categorical extremes. Finally, covariates included in the final models may account for differences in the conclusions. For example, we included pre-pregnancy BMI as a likely confounder whereas the Michels et al. analysis of birth weight did not [11].

There are a few aspects of our study that warrant a cautious interpretation of the findings. It is possible that underlying health conditions present at the time the blood sample was collected might influence the cellular constituency of the blood, influencing DNA methylation measured; however, these conditions (e.g., infection) are likely to be random rather than systematic. It should also be noted that variation in DNA methylation between birth and three years is likely a function of heritability as well as additional exposures occurring during these early years of life. While we were able to evaluate the impact of some of the non-heritable factors (e.g., exposure to smoke in the home, breast feeding), there are many postnatal exposures that we were not able to measure; whether any of these exposures confound observed associations is not known. Additionally, our pre-pregnancy maternal weight and height measures were self-reported, which may result in under-estimating weight and BMI and over-estimating height [37]. This potential misclassification is unlikely to be related to global DNA methylation levels (which are unknown to the study participants) therefore biasing the results to the null. Finally, global DNA methylation is an informative but limited biomarker whose clinical interpretation is not fully understood. It provides no indication of which genes are impacted, limiting our ability to interpret or predict potentially related health consequences.

## Conclusion

Global DNA methylation is a non-specific biomarker that is a broad indicator of early biological changes that may affect an array of functions in the offspring. The biologic implications of small changes in global DNA methylation are unknown. However, the observation that global DNA methylation levels persist within an individual from birth to age three supports the belief that factors that influence global DNA methylation may confer long-term effects. Therefore, the effect of these factors (including pre-pregnancy BMI) on global DNA methylation at birth and at three years deserves further study. Specifically, examining the DNA methylation of specific genes or regions of the genome that are influenced by pre-pregnancy BMI could contribute to the development of hypotheses about how the offspring of overweight women may be affected across the life course.

## Supporting Information

File S1 **Contains: Table S1** Comparison of those with and without cord DNA methylation and with and without three year DNA methylation. **Table S2** Factors predicting absolute value of the change in DNA methylation over time (change  =  |log-three year meth – log-cord meth|). **Figure S1 A–D:** Partial regression plots from multiple regression analysis that test the association between DNA methylation (cord or three year, as indicated) and pre-pregnancy maternal body mass index (BMI).
(DOCX)
