# Supplementary material for: Predictors and Consequences of Global DNA Methylation in Cord Blood and at Three Years
Source: PLoS One. 2013 Sep 4;8(9):e72824. doi: 10.1371/journal.pone.0072824 (PMC3762861; doi:10.1371/journal.pone.0072824)

**Table S1.** **Comparison of those with and without cord DNA methylation and with and without three year DNA methylation**

|  | Without Cord DNA Methylation | | With Cord DNA Methylation | | Without Three-year Methylation | | With Three-year DNA Methylation | |
| --- | --- | --- | --- | --- | --- | --- | --- | --- |
|  | N | % | N | % | N | % | N | % |
| **Maternal Education** |  |  |  |  |  |  |  |  |
| <HS | 228 | 40.28 | 100 | 35.97 | 45 | 39.82 | 55 | 33.33 |
| HS | 201 | 35.51 | 96 | 34.53 | 29 | 25.66 | 67 | 40.61 |
| >HS | 137 | 24.2 | 82 | 29.5 | 39 | 34.51 | 43 | 26.06 |
| **Marital Status** |  |  |  |  |  |  |  |  |
| Married/Cohab. | 412 | 73.44 | 206 | 75.18 | 87 | 78.38 | 24 | 24.62 |
| Unmarried | 149 | 26.56 | 68 | 24.82 | 119 | 73.01 | 44 | 26.99 |
| **Material Hardship** |  |  |  |  |  |  |  |  |
| Yes | 266 | 47.00 | 120 | 43.01 | 48 | 42.11 | 72 | 43.64 |
| No | 300 | 53.00 | 159 | 56.99 | 66 | 57.89 | 93 | 56.36 |
| **Public Assistance** |  |  |  |  |  |  |  |  |
| Yes | 507 | 90.05 | 255 | 91.73 | 100 | 88.5 | 155 | 93.94 |
| No | 56 | 9.95 | 23 | 8.27 | 13 | 11.5 | 10 | 6.06 |
| **Ethnicity** |  |  |  |  |  |  |  |  |
| Dominican | 355 | 62.83 | 193 | 69.18 | 82 | 71.93 | 111 | 67.27 |
| African American | 210 | 37.17 | 86 | 30.82 | 32 | 28.07 | 54 | 32.73 |
| **BMI** |  |  |  |  |  |  |  |  |
| Underweight | 35 | 6.18 | 14 | 5.02 | 5 | 4.39 | 9 | 5.45 |
| Normal weight | 268 | 47.35 | 125 | 44.8 | 51 | 44.74 | 74 | 44.85 |
| Overweight | 115 | 20.32 | 62 | 22.22 | 22 | 19.3 | 40 | 24.24 |
| Obese | 148 | 26.15 | 78 | 27.96 | 36 | 31.58 | 42 | 25.45 |
| **ETS** |  |  |  |  |  |  |  |  |
| Yes | 189 | 33.87 | 93 | 33.94 | 35 | 31.25 | 58 | 35.8 |
| No | 369 | 66.13 | 181 | 66.06 | 77 | 68.75 | 104 | 64.2 |
| **Former smoker** |  |  |  |  |  |  |  |  |
| Yes | 117 | 22.9 | 47 | 18.15 | 21 | 19.63 | 26 | 17.11 |
| No | 394 | 77.1 | 212 | 81.85 | 86 | 80.37 | 126 | 82.89 |
| **Sex** |  |  |  |  |  |  |  |  |
| Male | 222 | 49.33 | 130 | 46.59 | 52 | 45.61 | 78 | 47.27 |
| Female | 228 | 50.67 | 149 | 53.41 | 62 | 54.39 | 87 | 52.73 |
|  | **N** | **mean (SD)** | **N** | **mean (SD)** | **N** | **mean (SD)** | **N** | **mean (SD)** |
| **Maternal Height (in)** | 557 | 63.0 (4.0) | 277 | 63.7 (3.5) | 112 | 63.0 (3.8) | 165 | 64.1 (3.2) |
| **Pre-pregnancy weight** | 557 | 146.0 (37.3) | 270 | 151.1 (38.1) | 111 | 148.8 (36.3) | 159 | 152.7 (39.3) |
| **Weight gain** | 406 | 36.8 (71.0) | 252 | 34.3 (16.1) | 100 | 30.9 (15.8) | 152 | 36.5 (16.0) |
| **Maternal Age** | 521 | 25.2 (4.9) | 279 | 25.1 (4.8) | 100 | 2.3 (2.5) | 159 | 2.5 (2.1) |
| **Total PAH*** | 434 | 2.34 (2.03) | 268 | 2.44 (2.29) | 114 | 25.0 (4.7) | 165 | 25.1 (4.9) |

**Table S2.** Factors predicting absolute value of the change in DNA methylation over time (change = |log-three year meth – log-cord meth|)

|  | **Beta** | **Lower 95% CI** | **Upper 95% CI** |
| --- | --- | --- | --- |
| **Maternal Education** |  |  |  |
| HS (vs. no HA) | -0.213 | -0.921 | 0.496 |
| >HS (vs. no HA) | -0.660 | -1.465 | 0.145 |
| **Married/Cohab. (vs. unmarried)** | -0.005 | -0.751 | 0.741 |
| **Material Hardship** | 0.056 | -0.546 | 0.659 |
| **African American (vs. Dominican)** | 0.285 | -0.449 | 1.020 |
| **Prenatal ETS** | 0.097 | -0.638 | 0.832 |
| **Former smoker** | -0.588 | -1.323 | 0.146 |
| **Male (vs. female)** | 0.005 | -0.579 | 0.588 |
| **Multiparous (vs. nulliparous)** | 0.066 | -0.621 | 0.753 |
| **Pre-pregnancy BMI** | -0.056 | -0.108 | -0.004 |
| **Weight Gain** | -0.008 | -0.029 | 0.012 |
| **Maternal Age** | -0.022 | -0.099 | 0.055 |
| **Total PAH (ln-adjusted)** | 0.142 | -0.269 | 0.553 |
| **Breast Fed** | 0.029 | 0.000 | 0.057 |
| **ETS at 3 yrs** | -0.185 | -0.978 | 0.607 |
| **Cord methylation** | -0.011 | -0.239 | 0.218 |
| **Birthweight** | 0.000 | -0.001 | 0.001 |
| **Gestational age** | -0.091 | -0.333 | 0.151 |
| **R2** | 0.140 |  |  |
| **Adjusted R2** | 0.000 |  |  |

**Figure S1.** Partial regression plots from multiple regression analysis that test the

association between DNA methylation (cord or three year, as indicated) and pre-pregnancy maternal body mass index (BMI).

1. **All cord DNA methylation**

**
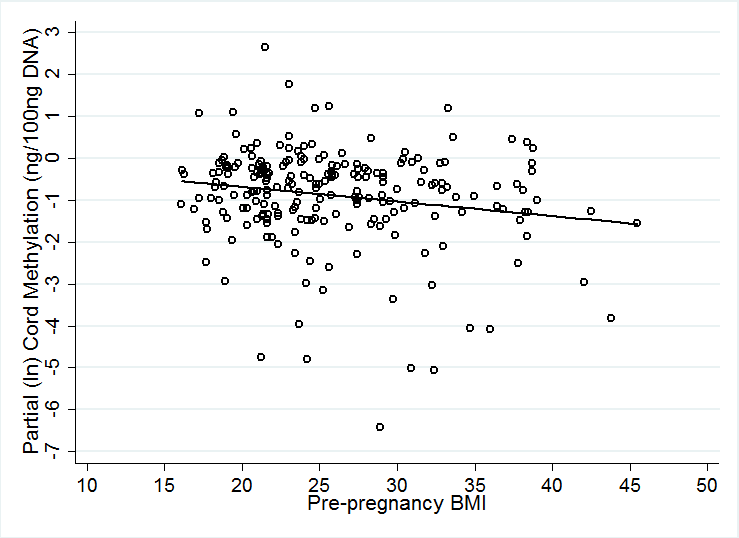
**

1. **Cord DNA methylation (subset with measured three year DNA methylation)**

**
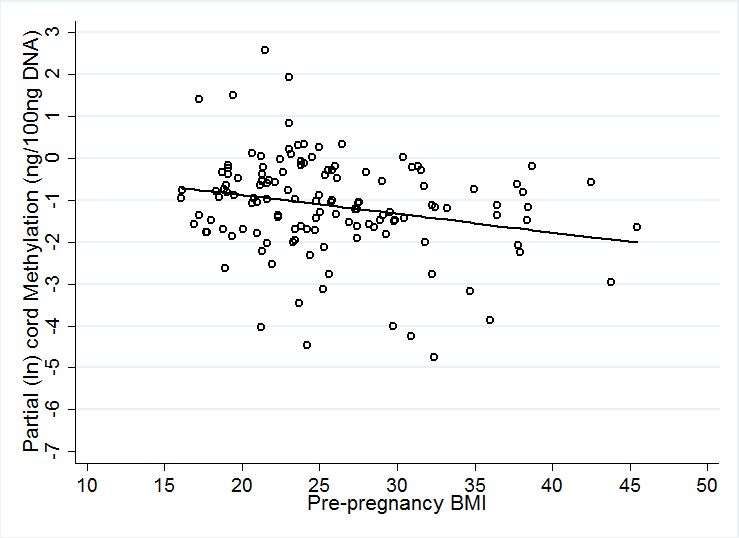
**

1. **Three year DNA methylation**

**
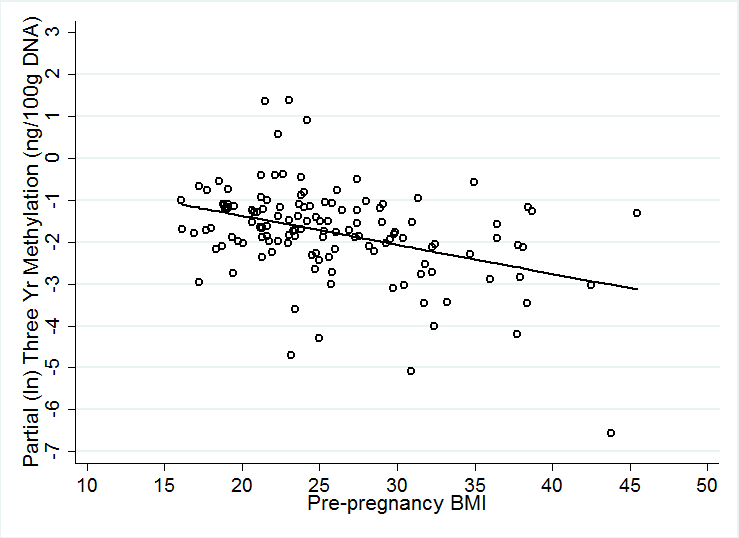
**

1. **Three year DNA methylation, adjusting for cord DNA methylation**


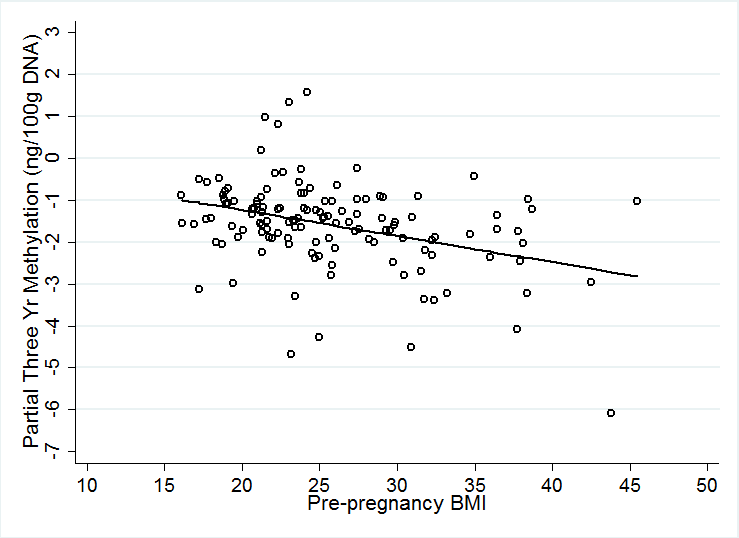

Supplement: File S1 — Contains: Table S1 Comparison of those with and without cord DNA methylation and with and without three year DNA methylation. Table S2 Factors predicting absolute value of the change in DNA methylation over time (change = |log-three year meth – log-cord meth|). Figure S1 A–D: Partial regression plots from multiple regression analysis that test the association between DNA methylation (cord or three year, as indicated) and pre-pregnancy maternal body mass index (BMI). (DOCX) [file pone.0072824.s001.docx]
